# Metacognitive Therapy Versus Cognitive Behavioral Therapy:A Network Approach

**DOI:** 10.3389/fpsyg.2018.02382

**Published:** 2018-11-30

**Authors:** Sverre Urnes Johnson, Asle Hoffart

**Affiliations:** ^1^Modum Bad Psychiatric Center, Vikersund, Norway; ^2^Department of Psychology, University of Oslo, Oslo, Norway

**Keywords:** metacognitive therapy, CBT, mlVAR, network approach, mechanisms

## Abstract

A network perspective on mental problems represents a new alternative to the latent variable perspective. Diagnoses are assumed to refer to a causal network of observable mental problems or symptoms (observables). The observable symptoms that traditionally have been considered indicators of latent traits (disorders) are taken to be directly related causal entities. Few studies have investigated how different therapies affect a network-structure of symptoms and processes. In this study, three anxiety symptoms, three depression symptoms and mechanisms in the form of cognitions, metacognitions, worry and threat monitoring were selected. The network structure over the course of therapy for metacognitive therapy (MCT) and Cognitive behavioral therapy (CBT) was investigated. It was hypothesized that worry, attention, and metacognition would be important nodes in MCT and that cognitions would be important in CBT. The data used in the analysis are from a RCT where 74 patients with comorbid anxiety disorders were randomized to either transdiagnostic MCT or disorder-specific CBT. Symptoms and mechanisms were measured every week. The data was analyzed using the multilevel vector autoregressive (mlVAR) model, which is currently the most developed method to analyze multivariate time series in multiple subjects and construct networks. The results indicate that there were different networks of symptoms and mechanisms in MCT and CBT. Central nodes in both treatments are worry and attention, however, the node of negative metacognitive beliefs about uncontrollability was more central in the MCT treatment. The results are consistent with predictions from the S-REF model.

## Introduction

Outcome in psychotherapy research is traditionally measured in relation to presence or absence of a disorder or severity of diagnostic symptoms. The disorder is assumed to be a latent entity, whereas the symptoms are viewed as indicators of this entity. The different indicators assumed to reflect the latent construct are rated and summarized in a total score. Mechanisms of change are treated as latent constructs and thus measured by total scores of relevant indicators. Thus, treatments are supposed to influence latent mechanisms that affect an underlying disorder manifested by specific symptoms (Borsboom and Cramer, 2013). However, there are several problems with this latent variable perspective (Borsboom, 2017; Hoffart and Johnson, 2017). First the latent variable perspective does not permit that symptoms cause each other. The symptoms are supposed to be caused by the underlying latent variable. In psychopathology, however, it makes sense that for example lack of sleep could lead to increased nervousness, or that lack of activity could lead to low mood. Thus, symptoms clearly influence each other. A new statistical approach called the network-approach takes this interdependence between symptoms into consideration (Borsboom and Cramer, 2013). The network approach conceptualizes symptoms as mutually interacting, often reciprocally reinforcing, elements of a complex network (Borsboom and Cramer, 2013). Thus different anxiety disorders do not exist as latent entities, but exists in the network of the symptoms. Each symptom could cause the release of others symptoms, and the comorbidity between disorders is explained by so called bridge symptoms or overlapping symptoms in the networks (Fried et al., 2017). This new approach opens up new questions regarding which and what kind of symptoms are the most central, so called centrality. Centrality-indices provide information about what kind of symptoms are most closely related to other symptoms, thus a promising target for interventions. In contrast, a latent disorders approach provides a sum-score that indicates the degree of anxiety or degree of depression. There are several differences between latent disorders models and network-models described elsewhere (Fried and Cramer, 2017; Bringmann and Eronen, 2018), but – for our purposes – the critical aspects are that modeling the data with network analysis gives new specific information about the relationship between symptoms, and that different treatments may activate different networks in similar patients.

An important purpose of therapy-models is to describe what maintains different symptoms or nodes in the network. These mechanisms, derived from theory, could also be called micro networks (Hoffart and Johnson, 2017). Both metacognitive therapy (MCT; Wells, 2009) and Cognitive behavioral therapy (CBT; Beck, 1976) specify micro-networks, however, the included variables are different. The origin of MCT can be traced to the early publications of the self-regulatory executive function model (S-REF; Wells and Matthews, 1994, 1996). The S-REF model consists of three interacting levels: a level of automatic and reflexively driven processing units; a level of attentionally demanding, voluntary processing; and a level of stored knowledge or self-beliefs (Wells and Matthews, 1996). The level of stored knowledge, or metacognitions, is involved in the development and maintenance of anxiety and emotional distress because these metacognitions give rise to the specific use of transdiagnostic strategies in the form of worry, rumination, and threat-monitoring. CBT originally developed by Beck (1967, 1976), is today an umbrella term of different therapies. In it’s traditional form schemas are thought to influence negative automatic thoughts that again drive specific symptoms. Thus, dysfunctional cognitions are thought to be crucial mechanism of change in CBT.

Several studies have investigated the role of metacognitions and cognitions in anxiety disorders (Smits et al., 2012; Johnson et al., 2018). Most of the studies have been conducted on at between-person level. Thus, it is investigated whether higher metacognitions or cognitions than the group mean predict anxiety. The reference-point is then the mean of all the patients. Therapists, however, are mainly interested in the within-person level, that is, if deviations from the persons own mean on a mechanism variable, are related to personal change on an outcome variable. Two studies have investigated if metacognitions predict anxiety on a within-person level, which requires repeated assessments points (Hoffart et al., 2018; Johnson et al., 2018). To our knowledge no studies have investigated MCT and CBT on a network of symptoms and mechanisms separating the within- and between person effects. Studies investigating network analysis have mainly used symptoms (Borsboom, 2017), even though there has been a call for networks that also include key mechanisms, such as cognition and metacognition (Jones et al., 2017).

The aim of this paper was to investigate the network structure of symptoms and mechanisms over the course of therapy in MCT and disorder-specific CBT separately. Since MCT and CBT emphasize different mechanisms it was hypothesized that worry, threat-monitoring and metacognition would be important nodes in MCT and that the cognitions would be important in CBT.

## Materials and Methods

The materials of this paper come from a randomized controlled trial (RCT) comparing MCT and CBT and are thoroughly described in two other papers (Johnson et al., 2017, 2018).

### Participants

Participants were referred to treatment at the Department of Anxiety Disorder at Modum Bad Psychiatric Center in Norway. Modum Bad is a specialized hospital running an inpatient treatment program for treatment resistant patients with anxiety disorders. The patients were referred because they had not benefited sufficiently from outpatient treatment. Recruitment was designed to be liberal using the clinical criteria for treatment used at the department. To be eligible for participation in the study, participants had to meet criteria for a principal DSM-IV disorder, exceeding 4 on the clinical severity rating (CSR), of PTSD, social phobia (SAD) or panic disorder with and without agoraphobia (PD/A). The Anxiety Disorders Interview Schedule (IV) (ADIS; Brown et al., 1994) was used to diagnose the patients. Further, participants had to have experienced failure of at least one structured psychological treatment, be 18 years of age or older, Norwegian speaking, and provide informed consent. Following the procedures at the department of Anxiety Disorders at Modum Bad, patients were excluded if (a) in a clinical context they would have required immediate treatment or simultaneous treatment that could interact with the treatment in unknown ways, (b) had current DSM-IV diagnosis of organic mental disorders, (c) clear and current suicidal risk, or (d) current substance abuse. All participants had to terminate the use of psychotropic medications before treatment, and were contacted before treatment to ensure that they were medication-free or had started discontinuation of medications. The study was approved by the Norwegian regional ethical committee (2013/209/REK South-East). All subjects gave written informed consent in accordance with the Declaration of Helsinki.

Patients were randomized to MCT or CBT stratified on their principal disorder.

All patients that started treatment, 74 participants (*n* = 38 CBT, *n* = 36 MCT) were included in the sample analyzed. Seven participants did not complete the treatment program, leaving 67 who completed all the treatment sessions (*n* = 33 in CBT, *n* = 34 in MCT). The average age was 42 (*SD* = 12.8), and there were 45 female and 29 male patients. The patients had on average 3.7 (*SD* = 1.6) diagnoses at the start of treatment, 41 % of the patients had a personality disorder. The duration of their anxiety problem was *M* = 16.1, *SD* = 11.8. A majority of the patients (80.5%) were either out of work or on a disability allowance, which indicates a sample with chronicity and poor level of functioning.

### Treatments

The number of sessions for completers were equivalent in both conditions (*M* = 9.4, *SD* = 1.7). The sessions in CBT lasted longer, due to the protocols of SAD and PTSD, which lasts 90 min. All therapists were trained in MCT or CBT, and the adherence and competence ratings of every session were above 4 on a scale from 0 to 6 (Johnson et al., 2017).

### Metacognitive Therapy

The MCT treatment consisted of a manualized treatment protocol for the generic MCT model (Wells, 2009). MCT is a process-oriented therapy. The protocol deemphasizes disorder-specific aspects, and focuses instead on challenging positive and negative metacognitions that drive the use of worry, rumination, threat-monitoring and coping behaviors, called the cognitive attentional syndrome (CAS), to regulate emotions.

### Cognitive Behavioral Therapy

Treatments in the disorder-specific CBT condition were the most extensively documented cognitive treatments of PD/A (Clark, 1986; Wells, 1997), of social phobia (Clark and Wells, 1995; Wells, 1997), and of PTSD by using prolonged exposure (PE) therapy (Foa et al., 2007). CBT is a content-based psychotherapy where the focus is on challenging the content of thought’s. Different catastrophic beliefs are thought to be central in different disorders. In PD/A thoughts about going crazy or loosing control are central, in social phobia thoughts about being embarrassed in front of others are key, and in PTSD thought that the world is dangerous and that the trauma is dangerous are central thoughts.

### Differences Between MCT and CBT

In MCT processes in the form of worry and the metacognitions that leads to the unhelpful thinking style is targeted. Thus, MCT also works with cognition, but on the level of metacognition. This can be exemplified with a patient who brings up a thought in session about being worthless. In CBT this thought could be taken for a possible schema about being worthless, and the reality of this belief could be tested. In MCT the statement about being worthless could be seen as either a trigger for rumination or an endpoint of rumination. The goal of the therapist is to challenge the dysfunctional metacognitions that drives the use of rumination. Further differences can be found in the use of exposure. In CBT, especially the PE-treatment, trauma-exposure is a critical component. In MCT exposure is not necessary, and reliving the trauma is not part of the treatment.

### Measures

In network analysis specific items are selected that captures the key processes that are under investigation. The two authors wanted to select central anxiety and depression symptoms as well as CBT-mechanisms and MCT-mechanisms. They independently selected the most appropriate items, and met to discuss whether there were any disagreements. There were none. The most relevant CBT-processes, MCT-processes and symptoms where then selected before the analysis. Three central anxiety symptoms were chosen from the Beck Anxiety Inventory (BAI; Beck et al., 1988), and three depression items from the Patient Health Questionnaire (PHQ-9; Kroenke et al., 2001). Two central cognitions were also chosen from the BAI, while three central processes in MCT where chosen from the CAS-1 (Wells, 2009). An overview of the items and measures can be found in Table 1.

**Table 1:** Abbreviation and meaning of the different nodes in the analysis.

Abbreviation	Meaning and measure
wor	Worry or dwelling on your problems (CAS-1)
att	Focusing attention on threatening things (CAS-1)
nmu	I cannot control my thoughts (CAS-1)
con	Fear of loosing control (BAI)
die	Fear of dying (BAI)
num	Numbness or tingling (BAI)
hea	Heart pounding/racing (BAI)
sha	Shaky/unsteady (BAI)
int	Little interest or pleasure in doing things (PHQ-9)
dep	Feeling down, depressed or hopeless (PHQ-9)
sle	Trouble falling or staying asleep, ore sleeping too much (PHQ-9)

### Statistical Analysis

The patient filled out the questionnaires every Monday during the course of therapy, giving a longitudinal dataset. The multilevel vector autoregressive (mlVAR) model is currently the most developed method to analyze multivariate time series in multiple subjects and construct networks (Epskamp et al., 2017). In time series data, consecutive responses are not likely to be independent (e.g., anxiety at one time point predicts anxiety at the next), thus violating a typical statistical assumption. The autoregressive (AR) part of mlVAR accounts for this time dependency within an individual by regressing a variable at time *t* on a lagged (measured at the previous time point, *t*-1) version of that same variable. The VAR model is a multivariate extension of the AR model. In VAR, variables are regressed on a lagged version of the same variable and all other variables of the multivariate set. Finally, the multilevel (ml) extension of the VAR allows the modeling of time dynamics across individuals. In mlVAR, each subject is assumed to have their own VAR model, and the VAR parameters vary randomly across individuals. In a mlVAR analysis three networks are estimated: a temporal network in which within-person effect predicts different nodes on the next time-point (lag 1), a contemporaneous network in which a node predicts another node at the same time-point, and a between-person network in which the overall score over the course of therapy are associated with other variables. The three network structures generated from our data are visualized through the R-package *qgraph* (Epskamp et al., 2012). The networks were calculated separately for MCT and CBT.

Centrality indices were calculated (Opsahl et al., 2010). These parameters indicate how central a node is in a given network. Outward degree is the sum of all outgoing connections, while inward degree is the sum of all incoming connections. Betweenness centrality takes into account both the direct and indirect connections of a symptom. Thus, a node with high betweenness centrality is a node that is located on many paths between other symptoms. It is thus an important node for how the network develops. Node-strength is the sum of all incoming and outgoing connections for the node.

### Model Assumptions

There are three central assumptions in using the mlVAR model. The first assumption is that the time intervals between two consecutive measurements are approximately equal. In this study the measures were included a week a part, every Monday, thus the assumption was fulfilled. The second assumption concerns stationary, indicating that the mean and variance of the series must stay unchanged. Stationary is often a problem in longitudinal dataset in clinical psychology, since most of the variables of interest are expected to change as a consequence of treatment. The variables were detrended according to the procedure outlined by Curran and Bauer (2011), and new variables were constructed consisting of the person-mean of all the measurements points as wells as the residuals from the detrending procedure. We used the Kwiatkowski-Phillips-Schmidt-Shin (KPSS) test for the null hypothesis that a time-series is level or trend stationary on the residuals from the detrending procedure (Kwiatkowski et al., 1992). The test was conducted separately for each of the patients and 11 variables per patient in each group using the R package tseries 0.10-43. The KPSS test indicated that the majority of time-series was trend (91%) and level (77%) stationary for MCT and CBT. The third assumption is the specific order of the model. We present only the results of the baseline models with lag-1 predictors included due to parsimony. In the network analysis a significance level of 0.05 for the individual effect was used. There was no correction for multiple testing, due to the exploratory nature of the study.

## Results

Positive relationship between symptoms is marked with green lines, while negative relationships are marked with red. The strength of the relationship between symptoms is represented by the thickness of the arrows in the figures. The thicker the arrow between two symptoms, and the closer the arrows are together in the figure, the stronger the relationship.

**FIGURE 1:**
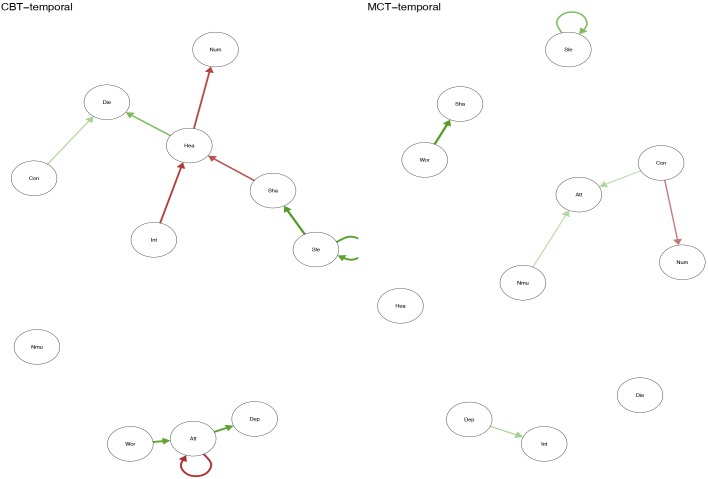
Temporal plots for CBT and MCT.

**FIGURE 2:**
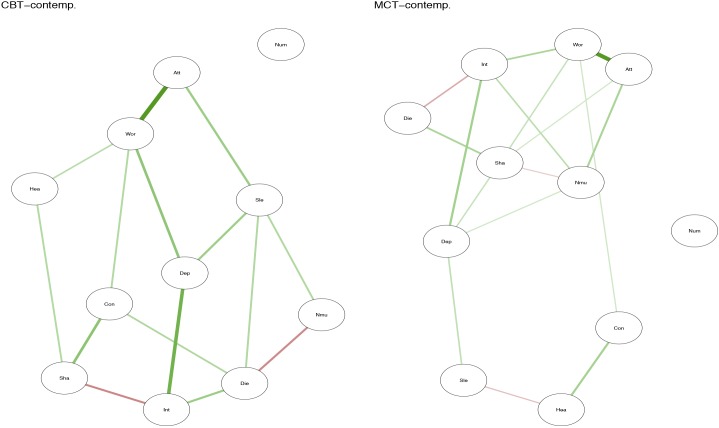
Contemporaneous plots for CBT and MCT.

**FIGURE 3:**
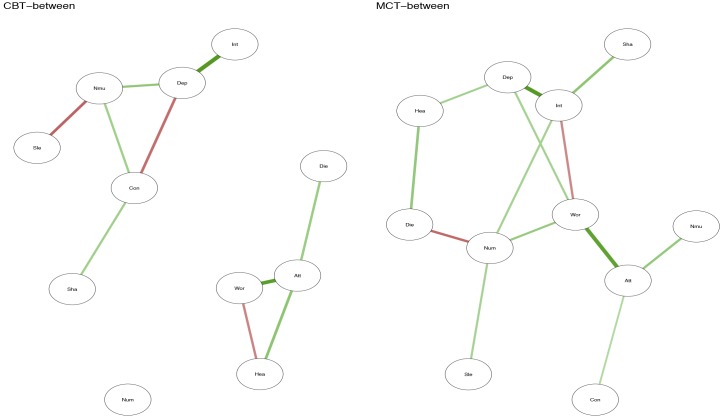
Between-person plots for CBT and MCT.

**FIGURE 4:**
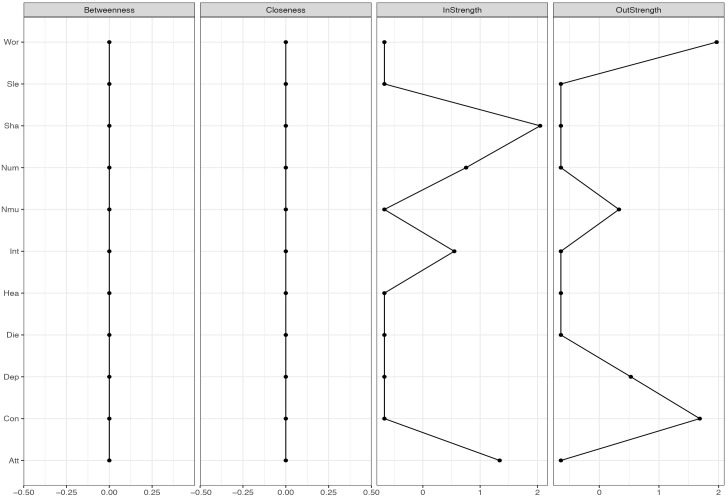
Centrality-plot for temporal effects in MCT. The higher the centrality index score the more central the symptom is in the network.

**FIGURE 5:**
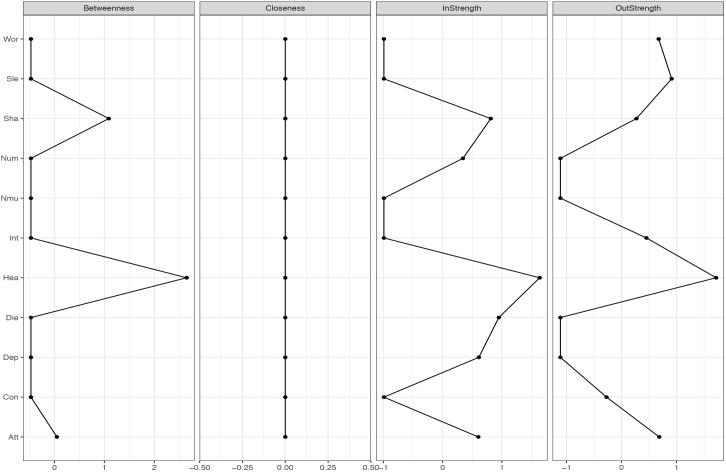
Centrality-plot for temporal effects in CBT. The higher the centrality index score the more central the symptom is in the network.

**FIGURE 6:**
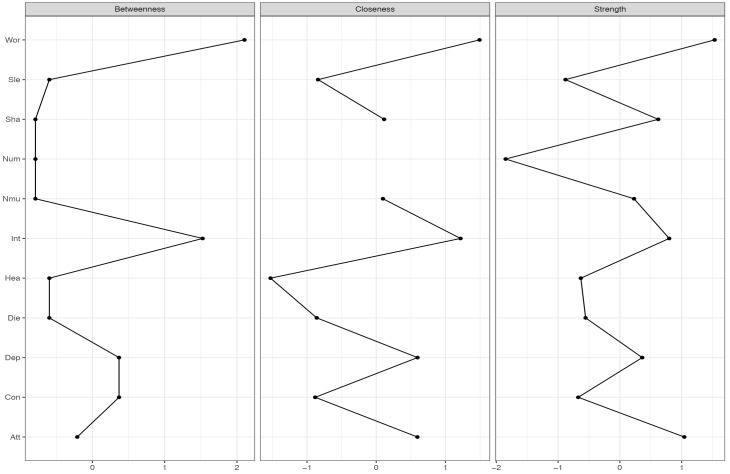
Centrality-plot for contemporaneous effects in MCT. The higher the centrality index score the more central the symptom is in the network.

**FIGURE 7:**
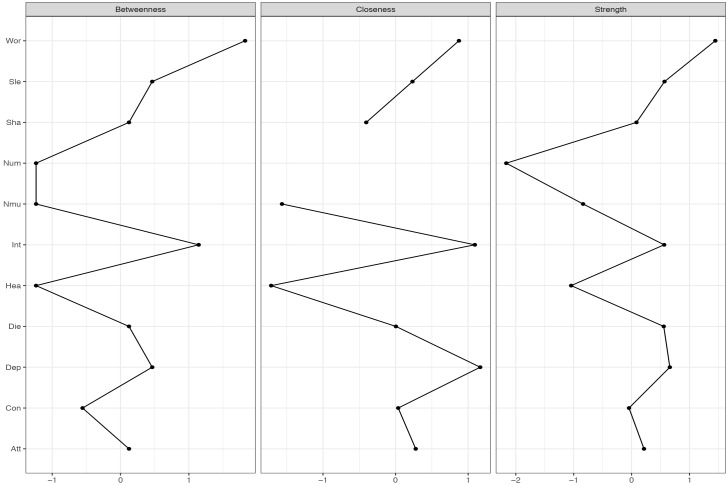
Centrality-plot for contemporaneous effects in CBT. The higher the centrality index score the more central the symptom is in the network.

The temporal network shows the averaged within-person effects from 1 week to the next. In the MCT network (see Figure 1, right side), the belief about uncontrollability of thoughts predicts threat-monitoring. Threat-monitoring, is also predicted by fear of losing control. Worry predicts the degree of feeling shaky. Thus, worry is a central node in the network, which is shown in the centrality indices in Figure 4. The CBT-network is more densely connected (see Figure 1, left side). The anxiety symptom of the heart pounding and raising is a central node. It is negatively predicted by the symptoms of shaky/unsteady and little interest. So higher levels of shaky/unsteady and little interest leads to less heart pounding at the next time point. Furthermore, the cognition fear of losing control predicts the cognition fear of dying, which, in turn, is predicted by heart pounding and racing. As shown in the centrality indices in Figure 5, worry, sleep and threat-monitoring are also central nodes.

The contemporaneous network captures the averaged within-person associations at the same measurement point, controlled for the lag-1 temporal effects. In the MCT network (Figure 2, right side), the belief about uncontrollability of thoughts is central as well as worry and threat-monitoring. The symptom of shakiness (sha) is also central in the network. This is evident in the centrality-plot in Figure 6. In the CBT plot (Figure 2, left side), worry and attention are still important nodes, but beliefs about uncontrollability of thoughts are less important. The centrality-plot is given in Figure 7.

The between-person network in Figure 3 shows the partial correlation between person-means on the 11 variables. In MCT, worry is again a central node, and worry is connected to threat-monitoring and threat-monitoring to the belief about uncontrollability of thoughts. The red line from worry to interest indicates that higher degree of worry is associated with less interest. Furthermore, a central symptom in the MCT-network was the feeling of numbness and lack of interest (see Supplementary Figure S1). In the CBT network the network is less connected, numbness is not a central symptom, but worry, feeling down and belief about uncontrollability of thoughts is (see Supplementary Figure S2).

## Discussion

The purpose of this study was to investigate the psychological networks in anxiety disorder patients receiving MCT versus CBT. The analysis indicated that the networks reflected the therapy form they received, especially with respect to the importance of nodes specified from the S-REF model. Across all three types of networks, worry and threat-monitoring were central nodes. Thus, worry and threat-monitoring are central in the maintenance of other symptoms or mechanisms in treatment-resistant anxiety disorder. It is previously argued that worry and threat-monitoring are important transdiagnostic mechanisms of change (Wells, 2009), but this results gives further empirical evidence for how these variables interact with other mechanisms and symptoms.

It was hypothesized that worry and metacognition would be important nodes in MCT. The networks for MCT indicated that for the three different networks metacognition, worry and attention to threat were densely connected. These results are consistent with the S-REF model, which predicts that metacognitions should affect the use of worry or threat-monitoring, as strategies for regulating low-level input or emotion (Wells and Matthews, 1996). It is also expected that the association between these variables should be strong, since these mechanisms are in the focus of treatment. Furthermore, lack of interest was a central symptom in MCT, indicating that targeting this symptom would also affect other symptoms. The clinical implications from the MCT-networks can be summarized by the centrality indices for the three different networks, especially the strength, which indicates how much change in a node will affect other nodes. On a between-person level, that is the overall means scores in therapy, reduction in worry and lack of interest was central in MCT. However, between-person relationships in longitudinal models can give limited information about how symptoms and processes develop over time (Bos et al., 2017). The temporal and contemporaneous networks, on the other hand, reflect within-person relationships and are therefore of particular relevance for therapeutic theories and the study of mechanisms of maintenance and change. This is because the mechanisms depicted in theories concern within-person relationships, that is, how change in a process variable in a given patient relates to change in an outcome variable during therapy. Consequently, it is also these two types of networks that provide clinical implications. In particular, nodes with high out-strength in the temporal network are targets for potentially effective interventions as changes in such nodes are likely to propagate through the network. It is evident in the MCT networks that worry, fear of losing control, and the meta-cognitive belief of uncontrollability of thoughts should be primary targets of treatment. These clinical implications are in accordance with MCT (Wells, 2009).

In the CBT networks the cognitions were associated on the temporal networks in association with specific symptoms. Thus, there is a relationship between catastrophic beliefs and symptoms, as would be expected from theory (Beck, 1976). Worry and attention were also central variables, which gives further support for the S-REF model. In the temporal network, there were also negative relationships between some bodily symptoms and between the depressive symptom disinterest and a bodily anxiety symptom (heart pounding/racing). These relationships probably reflect oscillation between reciprocally excluding emotional and bodily systems and are more a basis for therapeutic observation than for manipulation. The clinical implications from the CBT-networks can be summarized by the centrality indices. Threat-monitoring, worry, and sleep problems have high out-strength and should be targeted. Also heart pounding/racing has high out-strength. None of the cognitions have high out-strength, thus the clinical implications of which processes that should be targeted, is not in accordance with CBT-theory.

However, does the apparent influence of processes from the S-REF-model indicate that CBT therapists to a larger degree should target MCT-processes? Targeting the content of cognition using verbal reattribution (CBT technique), and proposing to leave the thoughts alone with detached mindfulness and postpone worry (MCT technique), could create confusion for the patient. In many ways the goal of the therapist in MCT and CBT is also incompatible. In MCT the goal is to change how patients respond to thoughts by changing metacognitions that drive the CAS. In CBT the goal is to change the content of thoughts. The finding that core processes, specified from the S-REF model, is the central nodes, rather implies the importance of targeting these processes in a metacognitive framework. It is previously shown that MCT was more effective then CBT in this treatment sample (Johnson et al., 2017). It is also evident that the two treatments have different networks, which may indicate treatment specificity. Thus, one possible explanation for the results in the RCT (Johnson et al., 2017) could be that MCT to a larger extent activated the association between worry, attention and metacognition.

Lack of interest being an important symptom in both MCT and CBT may be a bit surprising since the sample consisted of anxiety disorder patients. However, the present sample had high degree of comorbidity, with an average of 3.7 diagnoses (Johnson et al., 2017). Thus, the high centrality of lack of interest may be due to the treatment resistant aspect of the sample. Overall, the network-analysis across CBT and MCT gives a clear message about the importance of targeting worry and threat-monitoring in therapy.

Using network analysis allows for a more specified understanding of which symptoms and mechanisms that are crucial for therapeutic interventions. Specific predictions from therapy theories can be tested using network analysis on longitudinal data. This paper gives further evidence for the MCT-model, with the edges between the variables in the S-REF model being significant in both treatments. Network analysis could also be implemented in routine care situations. By having patients answer several questions repeatedly during a specific time-frame before treatment, an individual network can be made. The clinicians can then start to work directly on the most central symptom. Future research should investigate whether this specific use of network analysis could lead to larger treatment-effects.

Even though the paper has several strengths in the form of novel analysis and a new way to investigate treatments effects, several limitations should be acknowledged. In this paper the different networks were not tested against each other using significance tests, since that would likely be a power problem. The sample size is limited, even though normal for psychotherapy studies. To the authors knowledge there are no implemented packages in R to estimate stability and accuracy in longitudinal networks (Epskamp et al., 2018). In order to test if the results found with the mlVAR method could be replicated, the results should have been compared with a second validation dataset. However, no such data set was available at the time of the writing. Thus, future replications of the results are needed.

Analyzing psychotherapy data using a network approach is in its early stages, and it is therefore important to explore possible differences between treatments that could be tested in larger samples at a later stage. Furthermore in this study items from the BAI were used, thus fear of losing control and fear of dying might not be the most representative items for catastrophic beliefs. The items chosen for the concept of negative metacognitions about uncontrollability of thoughts such as, “I cannot control my thoughts,” is not representative for all aspects of metacognitions. Other aspects of metacognition like positive metacognitions, cognitive confidence, need for control and cognitive self-consciousness should also be investigated. In our models we used a *t*-1 lag, representing a week. Other relationship between the nodes could exist on other timeframes and should be investigated.

## Author Contributions

SJ performed the analysis and wrote the first draft of the manuscript. AH commented and assisted on the analysis and the writing of the manuscript.

## Conflict of Interest Statement

The authors declare that the research was conducted in the absence of any commercial or financial relationships that could be construed as a potential conflict of interest.
