# Supplementary material for: Metacognitive Therapy Versus Cognitive Behavioral Therapy:A Network Approach
Source: Front Psychol. 2018 Nov 30;9:2382. doi: 10.3389/fpsyg.2018.02382 (PMC6288670; doi:10.3389/fpsyg.2018.02382)
Supplement: Supplementary file 1 [file Data_Sheet_1.pdf]

Supplementary material

Metacognitive therapy versus Cognitive behavioral therapy: A network approach

Sverre Urnes Johnson [1*], Asle Hoffart [1, 2],

[1] *Modum Bad Psychiatric Center*
[2] *Department of Psychology, University of Oslo*

***Correspondence:**
Sverre Urnes Johnson
Sverre.johnson@modum-bad.no

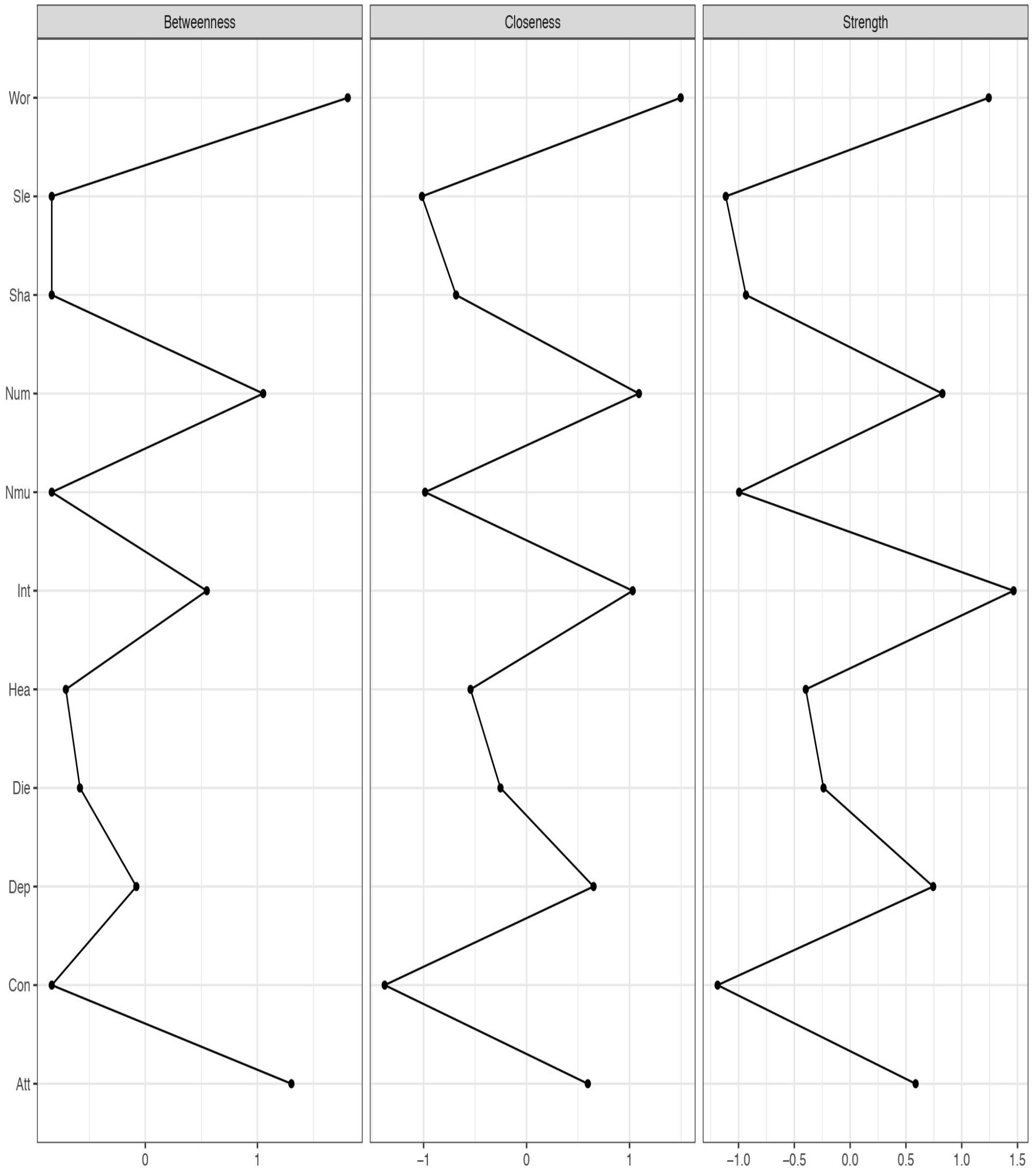

Figure S1. Centrality-plot for between-persons effects in MCT. The higher the centrality index score the more central the symptom is in the network.

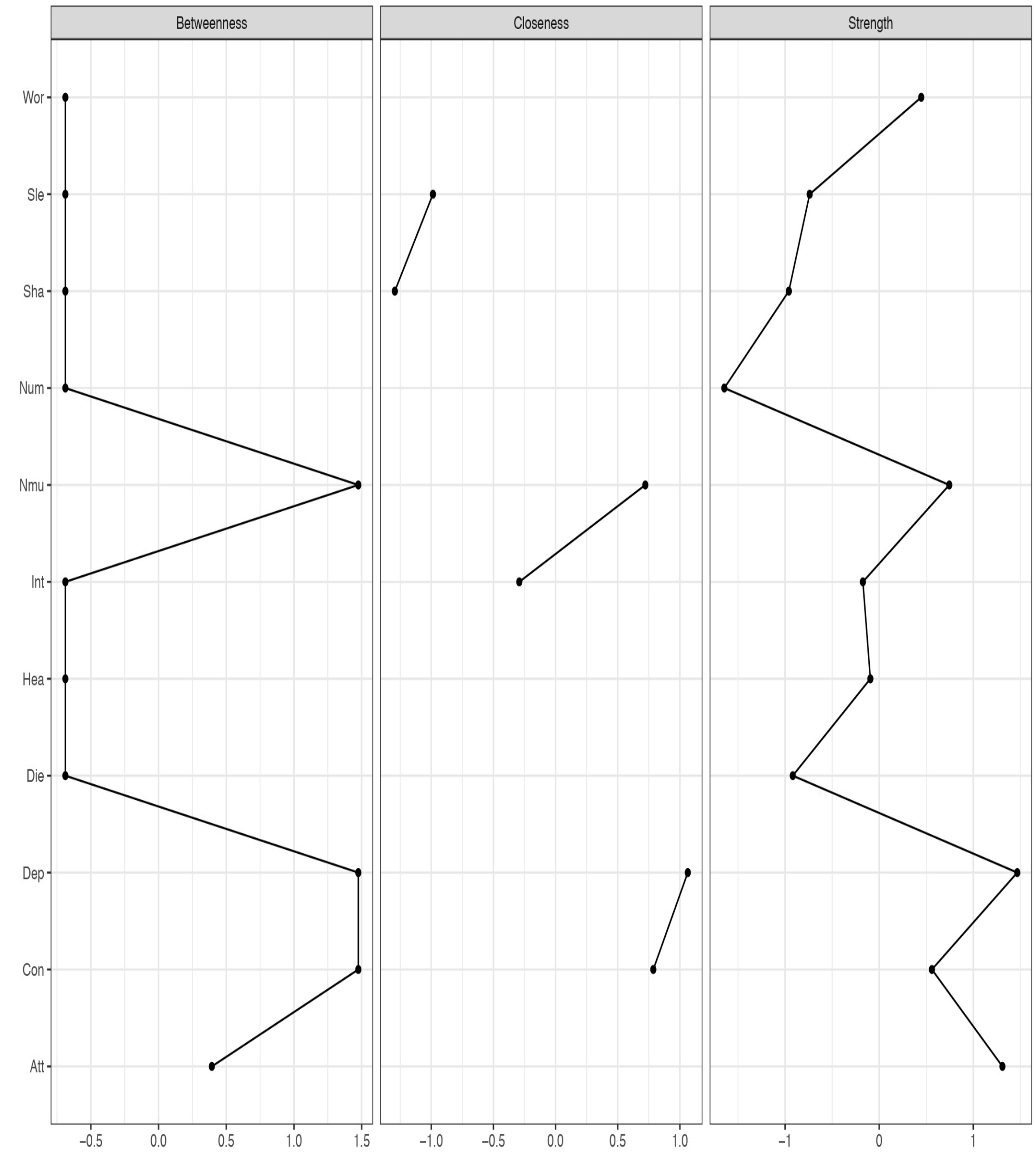

Figure S2. Centrality-plot for between-persons effects in CBT. The higher the centrality index score the more central the symptom is in the network.
